# Predictors of divorce and duration of marriage among first marriage women in Dejne administrative town

**DOI:** 10.1038/s41598-024-59360-5

**Published:** 2024-04-16

**Authors:** Nigusie Gashaye Shita, Liknaw Bewket Zeleke

**Affiliations:** 1 Department of Statistics, Debre Markos University, Debre Markos, Ethiopia; 2 Department of Midwifery, Debre Markos University, Debre Markos, Ethiopia

**Keywords:** Divorce, Binary logistic regression, Gompertz regression model, Dejne, Psychology, Oncology, Public health, Scientific data, Statistics

## Abstract

Divorce is a common occurrence in the marital lives of spouses. Consequently, numerous divorced spouses and their children face various social, economic, physiological, and health problems after breaking their marriage. This study aimed to identify the predictors of divorce and the duration of marriage. We conducted a community-based cross-sectional study among 423 randomly selected residents of Dejen Township in April 2020, of which only 369 respondents met the study inclusion criteria. We used structured questionnaires to collect data. The predictors of divorce and duration of marriage were analyzed using binary logistic regression and the Gompertz regression model, respectively. A *p* value less than 0.05 was used to express statistical significance. The prevalence of divorce was 21.14% [95% CI (19.01–23.27%)]. Half of these women broke up their marriage after 11 years. A high age difference (7 or more years) between spouses, an early marriage, infertility among women, the presence of third parties, women without formal education, women in the workforce, sexually dissatisfied women, women who did not live together with their husbands at the same address, partner violence, marital control behaviour of husbands, drug-abused husbands, spouses without children, and women who knew multiple sexual partners were the significant predictors of divorce. Partner violence, sexually dissatisfied women, women who made their own marriage decisions, marital control behaviour of husbands, women who did not live together with their husbands at the same address, drug-abused husbands and spouses without children were significant predictors of shorter marriage durations. In this study, the prevalence of divorce was high. Therefore, a community-based, integrated strategy is needed to minimize the divorce rate.

## Introduction

Marriage is an essential association for the establishment of the family institution. Through marriage, a man and a woman can fulfill their responsibilities as husband and wife together to build the family^1–3^. However, not all spouses can endure their marriage until the end, and even worse, they will face serious household conflict, thus causing a divorce^4^. Divorce is a final legal separation of married spouses; consult with individuals about their right to remarry under civil, religious, or other provisions, according to the laws of each country.

Globally, the trend of divorce increased with time^5^. A study from thirty-three sub-Saharan African countries indicated that 25% of first marriages ended in divorce, which ranges from 6.9 to 47.1% in the Congo^6^. A study in Ethiopia discovered that on average, 45% of all first marriages ended in divorce in 30 years, 28% of first marriages in the first 5 years, 34% in 10 years, and 40% within 20 years^7^. While the 2016 Ethiopian Demographic and Health Survey (EDHS) study revealed that one-fourth of married women (24.9%) ended in divorced^4^. Specifically, research conducted in Debre Birhan, Ethiopia, showed that about 41.7% of first-marriages were broken^8^. Similarly, a study conducted in the East Gojjam Zone, Ethiopia, showed that the general divorce rates per 1000 people in the Dejene and Gozamin words increased by 3.27% and 1.33%, respectively, from 2010 to 2011^9^.

Divorce is the right and decision of the two spouses in Ethiopia and is taken as a solution to abusive and violent marital life; however, it has several socioeconomic and health problems for divorced families^5,9–16^. For example, children from divorced families experienced mental and physical health problems in developed countries^17–19^, whereas the problem was more severe in low-income countries like Ethiopia^9,20^. Consequently, the results of divorce can be antisocial behavior, school dropout, participation in addiction, delinquent behaviour, theft, and immoral acts of conduct that can be developed in divorced families^9,21^ and increased risk of death of children of divorced parents compared to married parents^22^. Even if the remaining surviving children were more likely to be stunted, start school late, and have poor educational attainment comparable to children of married parents^23–27^.

The predictors of divorce were age at marriage, residence, education status, history of abortion, employment status, partner abuse, globalization, sexual satisfaction, and economic problems^4,6,8,10,28–32^. However, previous studies did not assess the influence of third party interference, the decision to marry, substance abuse, and the residence of the husband at the same address on divorce^4,6,10,28–32^. Furthermore, most of the divorce studies conducted in Ethiopia used descriptive statistics or binary logistic regression^4,9,14–16,21,27^.

Therefore, previous studies have not used Gompertz survival analysis to investigate predictors of divorce among women. However, the survival analysis of the Gompertz model (time-to-event (divorce)) offered more information than binary logistic regression (simply whether or not an event) occurred^33^. Survival analysis methods are the only recommended methods to handle outcomes (divorced) and censored observations where divorce did not occur in first-marriage women until the study period. Therefore, this study aimed to identify predictors of divorce and duration of marriage among first marriage women by binary logistic regression and Gompertz survival analysis, respectively. The findings of this study can help design a preventive strategy for divorce.

## Methods

### Study design, study area, and study period

A community-based cross-sectional study was conducted in the administrative town of Dejne in April 2020. Dejne is located in the east Gojjam zone of the Amhara region, Ethiopia, on the edge of the Blue Nile Canyon. It has an altitude and longitude of 50° 10′ N, 38°, 8′ E, and an elevation between 2421 and 2490 m above sea level.

### Inclusion and exclusion criteria

All married women willing to participate and who have lived in the town of Dejen for at least 6 months were included in this study; never married women were excluded from the analysis.

### Study population

The study population was all women who had ever married and lived in the town and who met the inclusion criteria for the study period, while the source of the population was a Dejne administrative town.

### Sample size determination and sampling methods

The sample size was calculated using a single population proportion formula by conducting a pilot survey. The proportion of divorce among first-marriage women in the pilot survey was 40%, and we used a 5% error margin (d) and a 5% level of significance. Then the initial sample size was calculated to be 369. That is $n=\frac{{Z}_{\alpha /2}^{2}*P(1-P)}{{d}^{2}}=\frac{1. 96*0. 4*0. 6}{{0. 05}^{2}}\hspace{0.17em}$= 369*.* Finally, we took into account a 15% non-response rate to determine the sample size. Subsequently, the sample size was 423.

**Figure 1:**
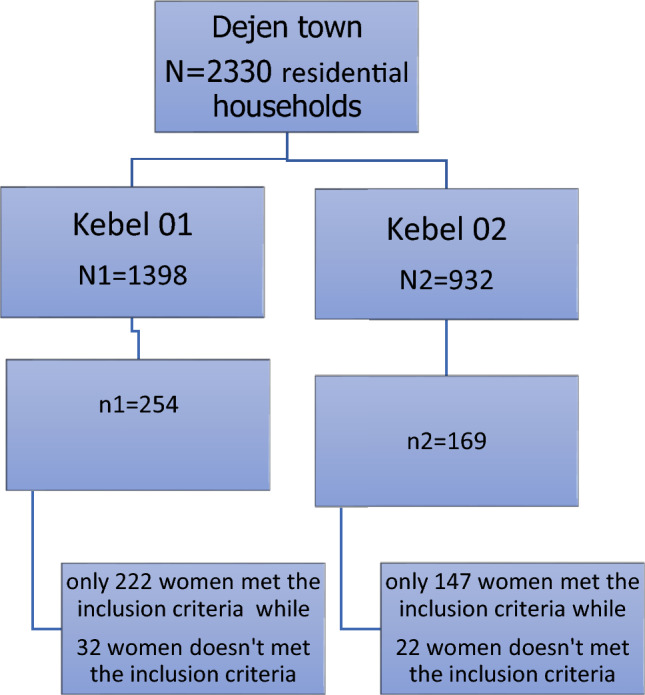
Schematic presentation for the sampling procedure of the study Predictors of divorce and duration of marriage among first marriage women in the administrative Town of Dejne, April 2020.

This sample size was proportionally allocated to each Kebele based on the number of households in each Kebel (Fig. 1). The town of Dejen is divided into two kebeles. We used this pre-arranged structure of the town to frame the current study.

Random sampling was utilized to choose study participants. Study participants were chosen from a list of households in the Dejen cities documented in the respective administrative units. We begin by randomly assigning a numeric code from one to two to each of the two kebles. Kebel 01 is 1, and Keble 02 is 2. We assign four-digit alphanumeric codes to each household in Kebele 01 and three-digit alphanumeric codes to each household in Kebele 02. For example, in Keble 01, there are 1398 registered households; therefore, the code for the first household was 0001, and the code for the last household was 1398. Kebele 02 had 932 households, with the first household having code 001 and the last having code 932.

Finally, among all households, randomly selected households have been selected using circular systematic sampling since ${N}_{l}\ne {k}_{l}*{n}_{l}$^34^, $where\;\;as\;\;l\;\;is\;\;number\;\;of\;\; kebels\;\;in\;\;Dejen\;\,twon = 1,2$. For example, the sample from Keble 01 was selected by applying circular systematic sampling. Circular systematic sampling consists of selecting a random number from 1 to 1398 using lottery methods and then selecting the unit corresponding to this random number. Subsequently, every unit is selected cyclically until a sample of units is obtained, k1 $=\frac{{N}_{1}}{{n}_{1}}$, which is the nearest integer to 5.504, which is 6. Here, the first household of houses selected is 11, and then the women corresponding to this random number. Then, by applying the following formal, we selected the remaining household, and after that, the participant was selected from the selected household.

$$\begin{aligned} & \begin{array}{*{20}l} {11 + j*6,} \hfill & {if\; 11 + j*6 \le 1398} \hfill \\ {11 + j*6 - 1398,} \hfill & {if 11 + j*6 > 1398} \hfill \\ \end{array} \\ & \;\;\;\;\;where\;\;as j = 1,2, \ldots ,253 \\ \end{aligned}$$

Similarly, Keble 02 samples were selected by applying circular, systematic sampling. Circular systematic sampling consists of selecting a random number from 1 to 932 using lottery methods and then selecting the unit corresponding to this random number. Thereafter, every unit is selected cyclically until a sample of units is obtained, k2 $=\frac{{N}_{2}}{{n}_{2}}$, being the nearest integer to 5.515, which is 6. Here, the first household of random selection is 017, and then we have chosen the first respondent from this household. Then, by applying the following formal, we selected the remaining household, and after that, the participant was selected from the selected household.

$$\begin{gathered} \begin{array}{*{20}l} {17 + j*6 ,} \hfill & {if 17 + j*6 \le 932} \hfill \\ {17 + j*6 - 932,} \hfill & { if 17 + j*6 > 932} \hfill \\ \end{array} \hfill \\ \;\;\;\;where\;\; as j = 1,2, \ldots ,168 \hfill \\ \end{gathered}$$

When multiple women met the inclusion requirements of the study, we used a lottery to choose one woman from each household. The household codes, which we published in the existing family archives, were removed as soon as the data collection process was completed.

### Data collection methods and data quality control

The study used primary data from the respondents sampled in the city who had ever married by building questionnaires. Data collectors were trained using a standard, structured and pre-tested questionnaire and supervised by the investigator. To minimize the errors in data collection that the enumerator may introduce, training was given for 2 days, and the questionnaire will be translated into Amharic to ensure that the enumerator understands the questionnaire very well.

### Study variable

Dependent variables were divorce among first-marriage women (yes or no) and the length of time (measured in years) from the date of the first-marriage formulation to the date of divorce (event or censored). Independent variables were demographic variables (age, status of women's education), economic variables (unemployment status), sociocultural variables (early marriage, age difference between spouses, interference from third parties, marital control behaviour of husbands, history of partner violence against women, decision to marry, residing with husband at same address, number of children of spouse, fertility status, number of sexual partners, habit of discussion with husbands) and biopsychological variables (substance abuse, sexual satisfaction).

### Operational definitions

Duration of marriage among first-marriage women means the length of time (measured in years) from the date of first-marriage formulation to the date of divorce (event or censored).

Censored means first marriage women who were not divorced until the data collection period, April 2020.

The event means first-marriage women who were divorced until the data collection period, April 2020.

Experience of partner violence if women reported any of the specified acts of physical, sexual, or emotional violence committed by their husbands^35^.

Living with a husband means that a woman lives with her husband at the same address^36^.

*Substance abuse is* a pattern of compulsive substance use marked by recurrent significant social, occupational, legal, or interpersonal adverse consequences, such as repeated absences from work or school, arrests, and marital difficulties.

*Substance use is* defined as the use of at least one substance (alcohol, khat, cigarettes, hashish, cannabis or heroin) during an individual’s lifetime to alter mood or behaviour^37^.

Women had marital control behaviour when their husband showed at least one of the following controlling behaviours: being jealous or angry if he spoke to other men, frequently accusing her of being unfaithful, not allowing her to meet her female friends, attempting to limit her contact with her family and insisting on knowing where she is at all times^35^.

### Ethics approval and consent to participate

Ethical approval was obtained from the Research Ethics Committee of the College of Natural and Computational Sciences (Debre Markos University) with protocol number NCS 4069/17/11. We confirm that all methods were carried out in accordance with relevant guidelines and regulations. Written informed consent was obtained from each participant prior to the interview. Participants who were unwilling to participate and wanted to withdraw at any stage of the interview had the freedom to do so without any restrictions.

### Data analysis

Descriptive statistics were used to describe the percentage and frequency of women. The Kaplan–Meier survival function and log-rank test were used to estimate and compare the survival experiences of first-marriage women among the different groups of participants, respectively. The predictors of divorce among first-marriage women were analyzed using binary logistic regression. Predictors of the duration of marriage among first-marriage women were analyzed using the Gompertz accelerated failure time model.

### Binary logistic regression analysis

We use binary logistic regression analysis to assess the predictors of divorce among first-marriage women. The specific form of the logistic regression model with unknown parameters, ${\beta }_{0}, {\beta }_{1}, \dots , {\beta }_{k}$ is

$$P_{i} = P\left( {y_{i} = 1|x_{i} } \right) = \frac{{e^{{\beta_{0} + \beta_{1} x_{i1} + \ldots + \beta_{k} x_{ik} }} }}{{1 + e^{{\beta_{0} + \beta_{1} x_{i1} + \ldots + \beta_{k} x_{ik} }} }}$$

The binary logistic regression model can be rewritten as:

1 $${\text{Logit}}\left( {P_{i} } \right) = {\text{log}}\left( {\frac{{P_{i} }}{{1 - P_{i} }}} \right) = X_{i}^{\prime } \beta .$$

where P_i_ = the probability of divorce for the ith respondent, Y_i_ = the observed marital status of the ith woman, and β is a vector of unknown coefficients. To estimate the values of the unknown parameters, we have used the maximum likelihood method of estimation.

### Accelerated failure time model

We use an accelerated failure-time model to assess predictors of the duration of marriage among first-marriage women. The general accelerated failure-time model has the form:

2 $$log{T}_{i}=\mu +{\alpha }_{1}{x}_{1i}+{\alpha }_{2}{x}_{2i}+\dots +{\alpha }_{p}{x}_{pi}+\sigma {\epsilon }_{i}$$

where $\mu$ is the intercept, ${x}_{1i}$ ,${x}_{2i}\cdots {x}_{pi}$ are the values of *p* explanatory variables of the ith woman. ${T}_{i}$ is denotes the observed failure time for the ith woman $(i=\mathrm{1,2},\cdots ,n)$, $\sigma$ is the scale parameter, and ${\epsilon }_{i}$ is denotes the ith observation error terms that have a standard probability distribution. Specifically, in this study, ${\varepsilon }_{i}$ is the best fit for the Gompertz distribution compared to other distributions because it has the smallest AIC and BIC compared to others (Table 1).

**Table 1:** Information criteria for the Parametric Survival Model on the predictors of duration of marriage among first-marriage women in the Administrative Town of Dejne, April 2020.

Distribution	AIC	BIC
Exponential	311.42	342.71
Weibull	269.35	304.55
Log logistic	277.8	320.82
Log normal	279.9	323.01
Gompertz	230.31	269.41

The hazard function for the Gompertz distribution can be written as follows:

3 $$h\left(t\right)=\lambda {{\text{e}}}^{\mathrm{\theta t}}$$

The parameter $\theta$ determines the shape of the hazard function. A positive value leads to a hazard function that increases over time.

### Model-building strategy

To build the models, we first performed a bivariate analysis for each of the explanatory variables and, based on statistical significance, identified the variables as candidates for multivariate analysis at the 0.2 level of significance^38^. As naturally different factors or variables do not operate separately, multivariate analysis helps to control for confounders and analyze the effects of a factor in the presence of other factors in the model. In multivariate analysis, variables at a significance level of 0.1 were included in the model^39^.

We used Akaike and Bayesian information criteria to select the appropriate models, and the model with the smallest AIC or BIC was considered the best fit^40,41^. In addition, we used the Hosmer-Lemshow test statistic to evaluate the goodness-of-fit of the model for binary logistic regression, and the better the model fit is, the smaller the difference between the observed and predicted observations.

## Results

### Characteristics of study participants

The analysis included a total of 369 participants who met the inclusion criteria. The median duration of marriage among first-marriage women (± interquartile range) was 11 years (± 13.5), ranging from 0.2 to 35 years. More than a quarter of women (32.2%) did not attend formal education, while more than three-quarters were orthodox followers (76.2%). About 30% of women reported experiencing some form of partner violence (emotional, physical, or sexual). Furthermore, approximately 30% of newly married women indicated that they had experienced at least one type of marital control behavior (Table 2).

**Table 2:** Cross tabulation of divorce among first-marriage women with predictor variables in Dejne Administrative Town, April 2020.

Variables	Category	Divorce		Total	Chi-square	DF	*P* value
		No (%)	Yes (%)				
Third person interference	Yes	230 (81.9)	51 (18.1)	281	6.314	1	0.012
	No	61 (69.3)	27 (30.7)	88			
Age at marriage	≤ 14 years	69 (71.1)	28 (28.9)	97	5.832	2	0.016
	15–17 years	105 (81.4)	24 (18.6)	129			
	≥ 18 years	117 (81.8)	26 (18.2)	143			
Age difference of husband and wife	≤ 6 years	156 (86.7)	24 (13.3)	180	12.842	1	0.000
	≥ 7 years	135 (71.4)	54 (28.6)	189			
Decision to marriage	My self	116 (81.1)	27 (18.9)	143	0.714	1	0.398
	Discussed with parents	175 (77.4)	51 (22.6)	226			
Number of sex partners	One	81 (84.4)	18 (18.2)	99	0.709	1	0.4
	≥ 2	210 (76.9)	60 (22.2)	270			
Sexual satisfaction	Not satisfied	87 (59.2)	60 (40.8)	147	56.577	1	0.000
	Satisfied	204 (91.9)	18 (8.1)	222			
Substance use	No	267 (82.7)	56 (17.3)	323	22.453	1	0.000
	Yes	24 (52.2)	22 (47.8)	46			
Residing with husband at the same address	Yes	252 (93.3)	18 (6.7)	270	126.43	1	0.000
	No	39 (39.4)	60 (60.6)	99			
Employment status	Unemployed	123 (85.4)	21 (14.6)	144	6.087	1	0.014
	Employed	168 (74.4)	57 (25.3)	225			
Marital control behavior	Yes	73 (67)	36 (33)	109	13.118	1	0.000
	No	218 (83.8)	42 (16.2)	260			
Habit of discussion with husbands	Absent	61 (42.2)	34 (35.8)	95	16.475	1	0.000
	Present	230 (83.9)	44 (16.1)	274			
Education status	No formal education	76 (67.3)	37 (32.7)	113	19.075	1	0.000
	Primary	68 (75.6)	22 (24.4)	90			
	Secondary and above	147 (88.6)	19 (11.4)	166			
History of Partner violence against women	Yes	56 (51.4)	53 (48.6)	109	70.106	1	0.000
	No	235 (90.4)	25 (9.6)	260			
Number of living children	Have no children	50 (62.5)	30 (37.5)	80	20.676	2	0.000
	1–2	148 (87.6)	21 (12.4)	169			
	> 2	93 (77.5)	27 (22.5)	120			
Fertility status	Infertile	35 (53.8)	30 (46.2)	65	29.618	1	0.000
	Fertile	256 (76.9)	48 (15.8)	304			

*Df* degree of freedom, *P value* probability value.

### Prevalence of divorce among first-marriage women

A current study found that 21.1% (95% CI 16.8–25.2%) of women who had ever been married had experienced divorce. Women who had a habit of discussing with their husbands had a lower rate of divorce than women who did not have a habit of discussing with their husbands (16.1% vs. 35.8%) (Table 2).

### Factors associated with divorce and duration of marriage

Age disparities of the spouse, age at marriage, fertility status, number of children, interference from third parties, educational status, sexual satisfaction, living with the husband at the same address, partner violence against women, marital control behaviour of the husband, husband who used substances and number of sexual partners of women who knew had a statistically significant association with divorce among first-marriage women at a level of significance of 0.05 (Table 3).

**Table 3:** Results of the binary logistic regression model with logit link for factors associated with divorce among first-marriage women in the Administrative Town of Dejne, April 2020.

Independent variable	Category	B	S.E	*P* value	AOR	95% CI for EXP(B)	
						Lower	Upper
Third-person interference	No	− 1.828	0.843	0.030	0.161	0.031	0.840
	Yes (ref)						
Age at marriage	≥ 18 years	− 3.609	1.149	0.002	0.027	0.003	0.258
	< 18 years (ref)						
Age difference	≤ 6 years	3.574	0.804	0.000	35.656	7.379	172.285
	≥ 7 years (ref)						
Decision to marriage	Discussed with parents	− 1.657	0.895	0.064	0.191	0.033	1.102
	Myself (ref)						
Sexual satisfaction	Satisfied	− 2.479	0.666	0.000	0.084	0.023	0.309
	Not satisfied (ref)						
Women husband used substance	Yes	6.047	1.500	0.000	422.974	22.383	7992.839
	No (ref)						
Residing with husband at the same address	No	4.413	0.889	0.000	82.480	14.430	471.460
	Yes (ref)						
Had marital control behavior of husband	No	− 2.396	0.660	0.000	0.091	0.025	0.332
	Yes (ref)						
Education status	Primary	− 3.721	1.112	0.001	0.024	0.003	0.214
	Secondary and above	− 1.290	0.774	0.096	0.275	0.060	1.254
	No education (ref)						
History of partner violence against women	No	− 2.090	0.688	0.002	0.124	0.032	0.476
	Yes (ref)						
Number of children	One or two children	− 7.167	1.318	0.000	0.001	0.000	0.010
	More than two children	− 6.467	1.392	0.000	0.002	0.000	0.024
	No children (ref)						
Fertility status	Fertile	− 3.887	0.797	0.000	0.021	0.004	0.098
	In fertile (ref)						
Number of sexual partners	Two or more	1.963	0.679	0.004	7.123	1.883	26.943
	One (ref)						
Constant		7.520	2.079	0.000	1844.777		

*Ref* reference, *B* estimated model coefficient, *S.E* standard error, *Df* degree of freedom, *P value* probability value, *CI* confidence interval, *Exp (B)* adjusted odds ratio (AOR).

The decision to marry, sexual satisfaction, living with the husband at the same address, partner violence against women, marital control behaviour of the husband, and the number of children had a statistically significant association with the duration of marriage among first-marriage women (Table 4).

**Table 4:** Results of the Gompertz accelerated failure time model for factors associated with duration of marriage among first-marriage women in Dejne administrative Town, April 2020.

Variable	Category	AHR	S.E	*P* value	95% CI for AHR	
					Lower	Upper
Decision to marriage	Discussed with parents	0.218	0.076	0.000	0.110	0.433
	Myself (ref)					
Sexual satisfaction	Satisfied	0.481	0.162	0.030	0.248	0.932
	Not satisfied (ref)					
Residing with husband at the same address	No	3.903	1.466	0.000	1.869	8.148
	Yes(ref)					
Had marital control behavior of husband	No	0.408	0.114	0.001	0.236	0.706
	Yes (ref)					
History of partner violence against women	No	0.490	0.168	0.037	0.250	0.959
	Yes (ref)					
Number of children	One or two children	0.277	0.086	0.000	0.151	0.508
	More than two children	0.140	0.048	0.000	0.072	0.274
	No children(ref)					
Number of sexual partners	Two or more	1.767	0.598	0.092	0.911	3.429
	One (ref)					
Constant		0.020	0.012	0.000	0.006	0.062
Gamma		0.171	0.019	0.000	0.134	0.208

*Ref* reference category, *AHR* Adjusted Hazard ratio, *S.E* standard error, *P* *value* probability value, *CI* confidence interval.

### Demographic variables

The study reveals that women who attended primary school (AOR 0.024, 95% CI 0.003–0.214) had lower chances of divorce compared to women who did not attend formula education.

### Economic variables

**Figure 2:**
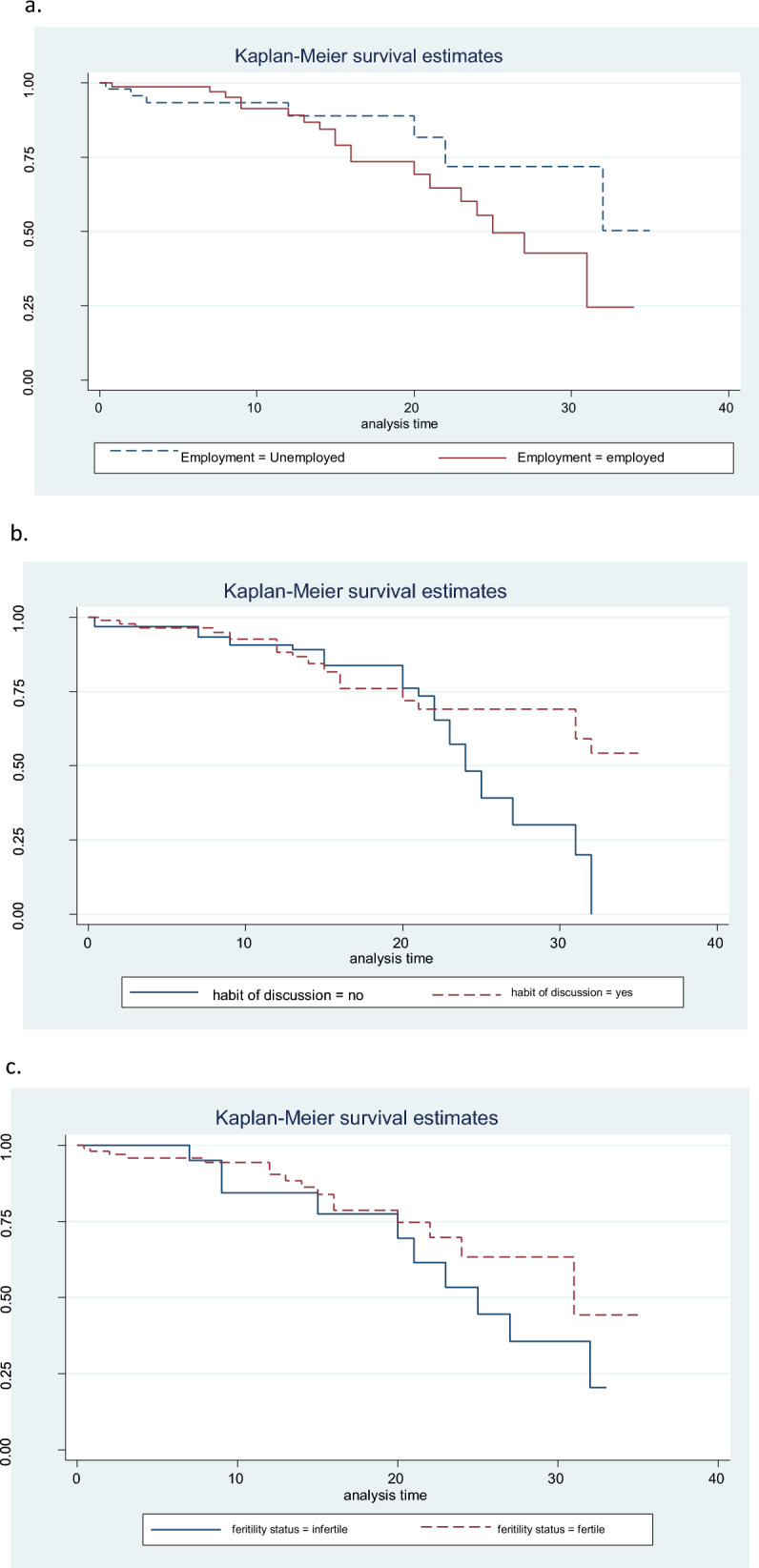
(**a**–**c**) Kaplan–Meier survival estimates on the survival experience of women's marriage for categorical variables in Dejne Administrative Town, April 2020.

This research indicated that the survival experience of first-marriage women had a statistically significant association with their employment status. Unemployed women have a longer survival experience in their first marriage than employed women (Fig. 2a–c).

### Socio-cultural variables

Women who were married at 18 years of age or older had a lower chance of divorce compared to women who were married at age < 18 years of age (AOR 0.027, 95% CI 0.003–0.258). Women who married husbands with an age difference of 7 years or more had a higher likelihood of divorce compared to those who married husbands with an age difference of 6 years or fewer (AOR 35.66, 95% CI 7.38–172.28). Women who did not have third party involvement in their marriage had a lower probability of experiencing divorce compared to women who had third party involvement [AOR 0.116, 95% CI (0.01–0.84)].

Women whose husbands did not exhibit controlling behavior had a lower likelihood of experiencing divorce than those whose husbands did exhibit controlling behavior (AOR 0.091, 95% CI 0.025–0.332). Similarly, women whose husbands did not exhibit controlling behavior had a lower risk of divorce among first marriage women than those whose husbands did exhibit marital controlling behavior [AHR 0.480, 95% CI (0.236–0.706)]. Furthermore, the study found that women who got married after discussing it with their parents had a lower risk of divorce among first-marriage women than those who decided to get married on their own decision [AHR 0.218, 95% CI (0.110–0.433)].

Women who did not report a history of some form of partner violence had a lower probability (AOR 0.214, 95% CI 0.032–0.476) of divorce compared to those women who reported a history of some form of partner violence. Similarly, women who did not inform of a history of some form of partner violence had a lower risk of divorce compared to those who reported a history of some form of partner violence [AHR 0.49, 95% CI (0.25–0.959)]. However, women who had the habit of discussing with their husbands had a longer survival experience of marriage among first-married women than those who had no habit of discussing with their husbands Fig. 2a–c.

Women who did not live with their husbands at the same address had higher odds of divorce compared to those who lived together at the same address [AOR 82.48, 95% CI (14.43–471.46)]. Women who had one or two children (AOR 0.001, 95% CI 0.0001–0.01) and those who had more than two children (AOR 0.002, 95% CI 0.0001–0.024) had lower odds of divorce compared to those women who had no children. In the same way, the risk of divorce for women who had one or two children and three or more children was 72.5% and 86% lower compared to women who had no children (AHR 0.275, 95% CI 0.151–0.508) and (AHR 0.14, 95% CI 0.072–0.274), respectively. Compared to infertile women [AOR 0.021, 95% CI (0.004–0.098)], those who were fertile had a lower chance of experiencing divorce. Similarly, fertile women also had a longer marriage survival experience among first-marriage women compared to infertile women (Fig. 2a–c). Women who knew two or more numbers of sexual partners had higher odds of divorce compared to women who knew only one number of sexual partners (AOR 7.123, 95% CI 1.83–26.943).

### Biopsychological factors

Sexually satisfied women had lower odds of divorce compared to sexually unsatisfied women (AOR 0.084, 95% CI 0.023–0.309). Similarly, the risk of divorce for sexually satisfied women was approximately 51.9% lower compared to sexually unsatisfied women (AHR 0.481, 95% CI 0.248–0.9322).

Women whose husbands used substances had higher chances of divorce compared to those women whose husbands did not use substances (AIR 422.97, 95% CI 22.38–7992.84). Similarly, the risk of divorce for women whose husbands used substances was 48.8% higher compared to those women whose husbands did not use substances [AHR 1.488, 95% CI (1.01–3.384)].

## Discussion

This research examined the predictors of divorce and the duration of marriage among first marriages in the Dejne administrative town, East Gojjam Zone.

The prevalence of divorce among women was 21.1% (95% CI 16.8–25.2%). This finding was consistent with the results of thirty-three sub-Saharan African countries and Ethiopia^4,6^. However, it is less than the study conducted in Gondar, Ethiopia (36.8%) and Bahir Dar, Ethiopia (46.5%)^42,43^. The difference observed between this and other studies could be the difference in the level of cities (this study was carried out in the administrative city of Woreda, whereas the previous study was carried out in regional metropolitan cities) and the difference in employment status; employed women have a higher prevalence of divorce^6,44^.

Women who completed primary school were more likely to divorce than those who did not attend formal school. This finding was consistent with previous investigations^4,6,45,46^. The possible reason for this study is that girls' education can raise women's marriage ages, and raising marriage ages would reduce divorce rates. Furthermore, educated women are less affected by external pressures when deciding on a marriage.

The findings of this research have shown that divorce of women has a statistically significant association with nine sociocultural variables: early marriage, the age difference between spouses, interference by third parties, marital control behaviour of husbands, history of partner violence against women, residing with the husband at the same address, number of children for the spouse, fertility status, and number of sexual partners.

Women without third-party influence were less likely to divorce than those with third-party interference. This study is in line with previous studies^8,9,42^. The possible reason for this study is that interference from third parties in the life of a married spouse is the main problem that hinders the continuation of marital relationships between spouses.

The chances of divorce were higher among women who were married at age < 18 years of age compared to women who were married at 18 or more years of age. This result is in line with prior findings^4,8,15,29,42,47^. The cause of this study could be a difference in maturity and marriage preparation. Furthermore, early married girls face sexual dysfunction in their later lives because they are more likely to have forced marital intercourse than those who marry later in life^48^.

Women who were married with an age difference of seven or more years between spouses had a higher risk of divorce than women who were married with an age difference of six or fewer years. This finding was consistent with previous findings^42^. The possible justification for this investigation is that the higher age difference between spouses is a cause of some difficulties, disagreements, and incompatible interests in their marital relationships, which can lead to divorce.

The odds of divorce were lower among women who had one or more children compared to those who had no children. Similarly, the chances of divorce were lower among women who were fertile compared to infertile women. This finding was in line with other studies^4^. The possible reason is that marriage is measured by the number of children they have in Ethiopia. Therefore, if marriage is not blessed by children, it is considered not to achieve its aim and a cause for divorce^49,50^.

Women who did not have marital control behavior of husbands had lower chances of divorce compared to women who did have marital control behavior of husbands. This result is similar to previous findings^8,47^. Women who did not have a history of partner violence were less likely to divorce than those who did. Other research has validated this finding^4,6,51^. The study could be justified by the fact that divorce is a last resort to deal with spousal violence if other techniques, such as correcting the causes of violence or living with the problem^52^.

The results of this study also revealed that divorce has a significant association with biopsychological factors such as substance abuse and sexual satisfaction. Women whose husbands were used to substance abuse had higher odds of divorce compared to women whose husbands were not used to substance abuse. The possible justification for this study is that most substance abusers are unable to perform what is expected of them. In addition, other side effects of substance abuse have also created many problems in their marital lives.

Sexually satisfied women had lower odds of divorce compared to those who were not sexually satisfied. This study is consistent with other studies^8,42,53^. The possible justification for this study is that sexual satisfaction contributes to healthy relationships and individual well-being. In addition, sexuality is an integral part of human life.

Unemployed women have a longer experience of first marriage than employed women. The finding of this study was similar to other studies^4,6,44^. The possible justification for this study is that unemployed women fear divorce due to their economic insecurity, which would increase the duration of marriage. Consequently, many unemployed women may have a problem in their marital life compared to employed women.

The risk of divorce for women who did not announce a history of some form of partner violence was 51% lower compared to those women who reported a history of some form of partner violence. Similarly, the risk of divorce for women whose husbands had no marital control behavior was 59.2% lower compared to those women whose husbands had marital control behavior. This result agrees with previous findings^8,54^. The likely explanation for the study is that the trust, respect, understanding, and equity relationship values they obtained from their husbands were critical to marital pleasure^55^, which may result in a longer marriage.

The risk of divorce for sexually satisfied women was approximately 51.9% lower compared to women who were not sexually satisfied. This result is in line with prior findings^8^. The study could be motivated by the fact that monotony, routine, and lack of variation hurt sexual activity in long-term relationships^56,57^.

One of the strengths of the study is its use of advanced statistical methods. We used the Gompertz-accelerated failure time model to evaluate the predictors of marital duration among first-marriage women (time-to-event analysis). Additionally, binary logistic regression is used to assess the predictors of divorce. The application of advanced statistical methods may yield a valid and efficient inference. The limitation of this study is that it used a cross-sectional study design, making it impossible to determine a cause-and-effect association between factors and divorce among women. The study collected data from women who broke their marriage before the study period, since recall bias could be the other limitation of the study.

## Conclusions and recommendations

The proportion of divorce among women was high in the administrative town of Dejne. The median duration of the marriage was 11 years. Improving women's education, cohabitation with husbands at the same address, preventing early marriage, addressing third-party interference, avoiding significant age differences (seven or more years), stopping substance abuse, averting marital control behavior by husbands, and combating partner violence against women would reduce the divorce rate in Dejne town. Sexual dissatisfaction, knowing more than one sexual partner, and being childless increased the prevalence of divorce.

Open communication marriage decisions, living with husbands at the same address, preventing substance abuse by husbands, avoiding marital control behaviour of husbands, and addressing partner violence against women would decrease the risk of divorce. Sexual dissatisfaction and being childless increased the risks of divorce.

This study suggests that increasing women's education, particularly primary school, could help reduce the rate of divorce among women. To reduce the divorce rate, family counsellors, social workers, and other help professionals should raise awareness of the effects of early marriage, third party interference, substance abuse, age disparities between spouses, not living together at the same address, marital control behaviour of husbands, lack of discussion, partner violence against women, and women who know multiple partners. Spouses should have a habit of discussing, especially sexual matters.

## Data Availability

The data sets analyzed in this study are available from the corresponding author on reasonable request.
